# Removal of Congo Red from Aqueous Solution by Anion Exchange Membrane (EBTAC): Adsorption Kinetics and Themodynamics

**DOI:** 10.3390/ma8074147

**Published:** 2015-07-08

**Authors:** Muhammad Imran Khan, Shahbaz Akhtar, Shagufta Zafar, Aqeela Shaheen, Muhammad Ali Khan, Rafael Luque, Aziz ur Rehman

**Affiliations:** 1 Department of Chemistry, the Islamia University of Bahawalpur, Bahawalpur 63000, Pakistan; E-Mails: raoimranishaq@gmail.com (M.I.K.); akhtar.shahbaz15@gmail.com (S.A.); shagutazafar25.sz@gmail.com (S.Z.); aqeelashaheen1@gmail.com (A.S.); 2 Fujian Institute of Research on Structure of Matter, Chinese Academy of Sciences, Fuzhou 350002, China; E-Mail: malichem92@fjirsm.ac.cn; 3 Departamento de Universidad de Córdoba, Edificio Marie Curie, Ctra Nnal IV-A, Km396, Córdoba E14014, Spain; E-Mail: q62alsor@uco.es; 4 School of Chemistry and Material Science, University of Science and Technology of China, Hefei 230026, Anhui, China; E-Mail: raoimranishaq@gmail.com

**Keywords:** adsorption, congo red, anion exchange membrane, Kinetics, thermodynamics

## Abstract

The adsorption behavior of anionic dye congo red (CR) from aqueous solutions using an anion exchange membrane (EBTAC) has been investigated at room temperature. The effect of several factors including contact time, membrane dosage, ionic strength and temperature were studied. Kinetic models, namely pseudo-first-order and pseudo-second-order, liquid film diffusion and Elovich models as well as Bangham and modified freundlich Equations, were employed to evaluate the experimental results. Parameters such as adsorption capacities, rate constant and related correlation coefficients for every model were calculated and discussed. The adsorption of CR on anion exchange membranes followed pseudo-second-order Kinetics. Thermodynamic parameters, namely changes in Gibbs free energy (*∆G°*), enthalpy (*∆H°*) and entropy (*∆S°*) were calculated for the adsorption of congo red, indicating an exothermic process.

## 1. Introduction

Improper treatment and disposal of dye-contaminated wastewaters from textile, dyeing, printing, ink and related industries have originated serious environmental concerns worldwide [1,2,3,4,5,6,7,8]. Certain quantities of dyes are irremediably lost in the manufacturing process and their effluents have to be carefully treated prior to discharge to minimize environmental damage. As regulations become stringent, complicated and multi-step treatments for dye wastewater are increasingly required, with dyes removal being key steps to consider. Several methods have been proposed for dye removal including biological treatments [7,9,10] coagulation/flocculation, ozone treatments [2,7] advanced chemical oxidation and photocatalytic processes [8,11,12] membrane technologies [1,2,3,13] and adsorption [2,3,4,5,6,7,14,15,16,17]. Among these, adsorption is often considered as a simple and efficient method [15,17]. Adsorbents tested in the literature included natural or synthetic products such as zeolites, activated clays and carbons, chitosan beads, cellulosic and polymeric resins, modified rice husk and cross-linked starch, palm kernel fiber, red mud and others [14,15,16,17,18,19,20,21,22,23,24,25,26,27,28,29]. Their price and efficiency varies from one adsorbent to another.

In recent years, commercial anion exchange resins have been shown to possess excellent adsorption capacities and demonstrate efficient regeneration properties for the removal and recovery of reactive dyes [4,30]. Anion exchange resins, generally in particle form, entail certain disadvantages when employed in packed-bed operations, which include slow pore diffusion, low accessible flow rates, high pressure drop and flow channeling. To circumvent the above limitations, anion exchange membranes (instead of resin particles) have been successfully implemented to remove anionic reactive dyes Cibacron blue 3GA and Cibacron red 3BA from aqueous solution [31]. The macroporous membrane system can, not only remove the technical problems of packed-bed operations, but also exhibits an improved simplicity and potential to scale-up via simple membrane-stacking and/or the use of a large membrane area. Ion exchange membranes consequently emerged as a potentially useful alternative adsorbent for industrial applications. However, fundamental studies are still required to better understand the adsorption mechanisms of some complex dye systems onto membranes (Kinetics, thermodynamics, *etc*.) in order to implement the use of such technologies at industrial scale.

**Figure 1:**
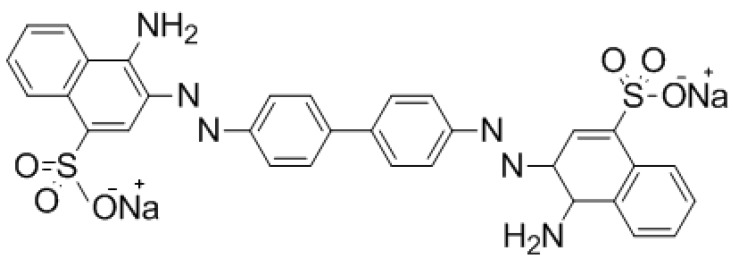
Structural formula of congo red (CR) dye.

This contribution has been aimed to provide a fundamental study of the use of commercial anion exchange membranes (EBTAC) to remove anionic dyes (congo red (CR) (Figure 1)) from aqueous solutions to mimic wastewater treatment experiments/conditions. Several parameters of the adsorption of the dye from aqueous solutions onto the membrane have been studied, namely the effect of contact time, membrane dosage, temperature and ionic strength in batch mode. Kinetic models such as pseudo-first-order and pseudo-second-order, liquid film diffusion and Elovich model were applied to all experimental data and Kinetics and equilibrium parameters were measured and compared. Thermodynamic parameters namely changes in free energy, enthalpy and entropy for CR adsorption were determined at different temperatures. It is worth pointing out that congo red, based on benzidine, is banned in several countries because of health concerns and its commercial use is limited.

## 2. Results and Discussion

### 2.1. Effect of Operational Parameters

Herein, the influence of operational parameters such as contact time, membrane quantity, ionic strength and temperature on the removal of CR dye from aqueous solution has been discussed. Their details are given below.

#### 2.1.1. Effect of Contact Time

**Figure 2:**
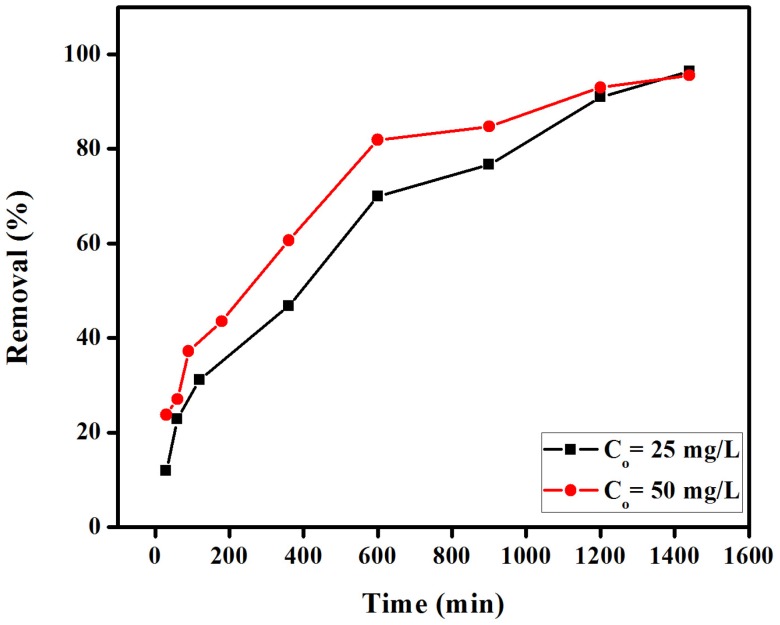
Effect of contact time on the removal (%) of congo red using anion exchange membrane EBTAC.

The effect of contact time on the percentage removal of CR from aqueous solution using EBTAC was investigated keeping initially constant the membrane dosage (0.1 g), dye concentrations (25 mg/L and 50 mg/L), volume of solution (40 mL) and stirring speed (120 rmp) at room temperature (Figure 2) Results indicate that the uptake of CR dye was very fast at the beginning (first 3 h) and then continued to increase until reaching equilibrium after 22–24 h (almost complete removal of the dye). Such behavior is typical from the presence of several adsorption sites on the membrane surface in the initial stage of reaction, which gradually gets saturated with the dye at increasing contact times. Repulsive forces between solute molecules on the solid and bulk phase can also contribute to the observed moderate rates of adsorption after the first 2–3 h.

#### 2.1.2. Effect of Membrane Quantities

**Figure 3:**
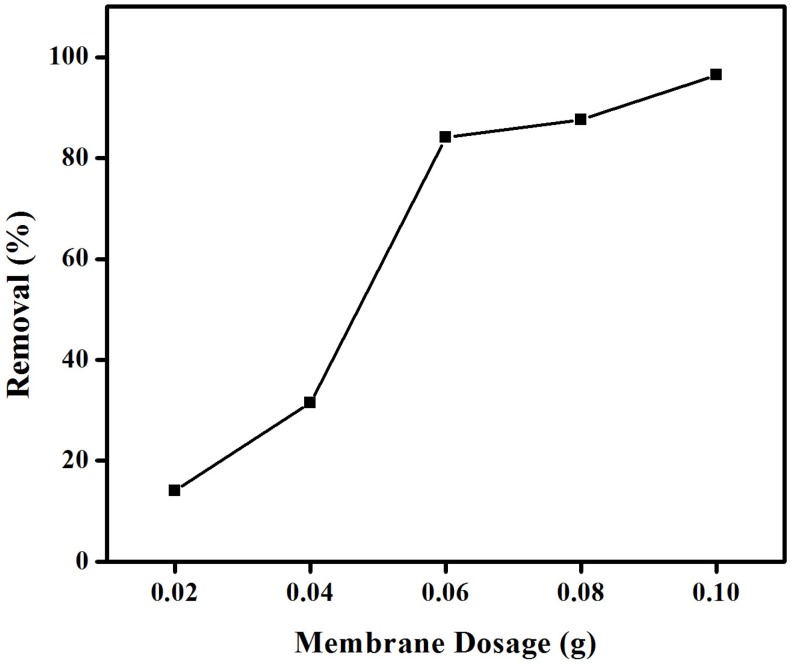
Effect of membrane dosage on the removal (%) of congo red using an anion exchange membrane EBTAC.

The influence of membrane quantities on the percentage removal of CR from aqueous solution was studied keeping constant the remaining parameters, with results presented in Figure 3. As expected, the percentage removal of CR remarkably increases at increasing the membrane dosage from 0.02 to 0.1 g (*ca*. 14% to almost 88% CR removal). This increase in adsorption was attributed to the increase in the number of available sorption sites on the surface of the anion exchange membrane. The removal of CR was rapid in the initial stage, remaining almost unchanged with a further increase (over 0.06 g) in the quantity of membrane material (Figure 3). In any case, CR was almost completely removed (>98%) at membrane quantities of 0.1 g. Consequently, 0.1 g was selected as optimum amount and used in further experiments in adsorption optimization experiments. The observed two stage-dependent adsorption behavior has also been previously reported in the literature [32].

#### 2.1.3. Effect of Ionic Strength

**Figure 4:**
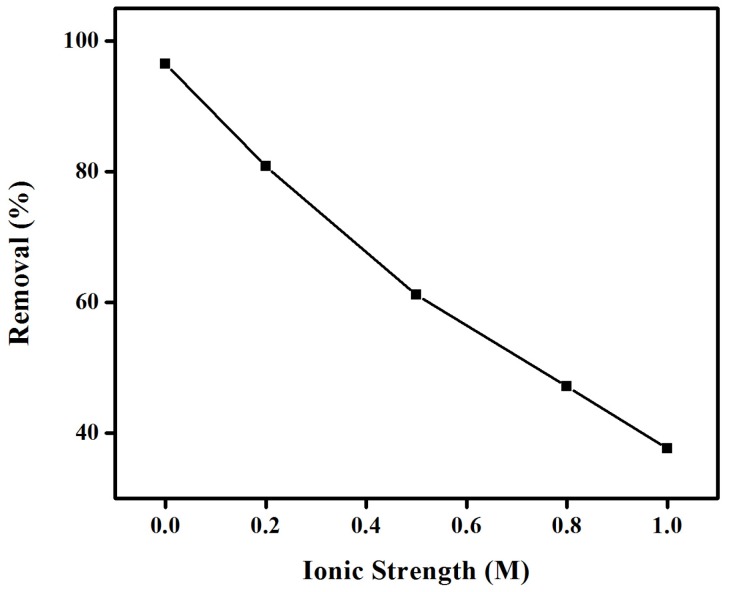
Effect of ìonic strength on the removal (%) of congo red using EBTAC.

The ionic strength of the solution is an important parameter that controls both electrostatic and non-electrostatic interactions between dyes and membrane surfaces. The effect of ionic strength on the removal of CR from solution was subsequently investigated by the addition of different quantities of common salt to the dye solution. Results depicted in Figure 4 clearly pointed out a decrease in the removal of CR at increasing salt concentrations, with a remarkable 38% obtained (as compared to an original 97%) after increasing NaCl concentration from 0 to 1 M. These results may relate to the competition between CR anions and Cl^–^ (from NaCl) for the active sorption sites, in good agreement with previous reports [33]. Most importantly, the efficiency of adsorption of the dye is also significantly affected by the ionic strength of the aqueous liquor. This is disadvantageous as salting-out is commonly used industrially to separate dyes from solution.

#### 2.1.4. Effect of Temperature

**Figure 5:**
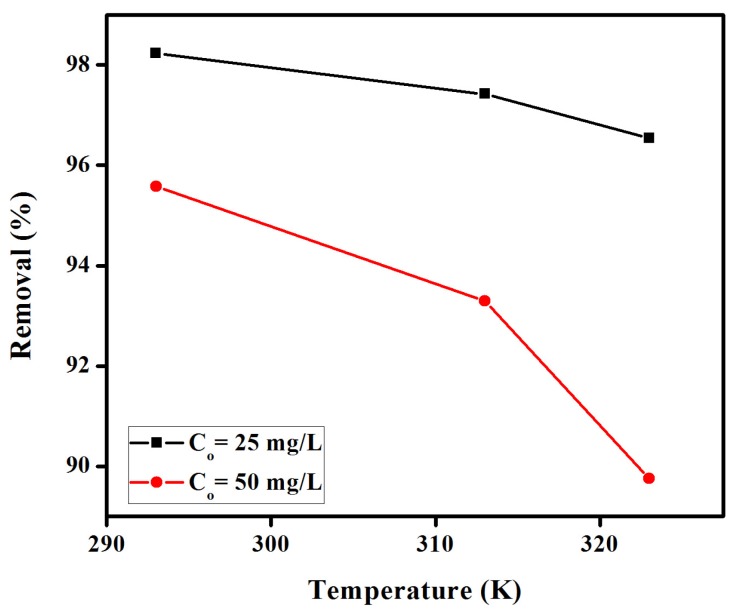
Effect of temperature on the removal (%) of congo red by anion exchange membrane EBTAC.

The effect of temperatures on the removal of CR from aqueous solutions was further studied keeping contact times, membranes dosage, stirring speed, solution volume and concentrations (25 to 50 mg/L) constant. Results shown in Figure 5 indicate a slight decrease in the adsorption of CR at increasing temperatures, although this parameter was not found to have such as significant influence in CR adsorption as compared to ionic strength or quantities of employed adsorbant. Such decrease (98% to 96% as well as 96% to 90%) was observed with increasing temperatures from 292 to 323 K for CR initial concentrations of 25 mg/L and 50 mg/L, respectively. These results also pointed out a potential exothermic process for CR adsorption on anion exchange EBTAC membranes.

### 2.2. Adsorption Kinetics

#### Kinetic Model Studies

**Figure 6:**
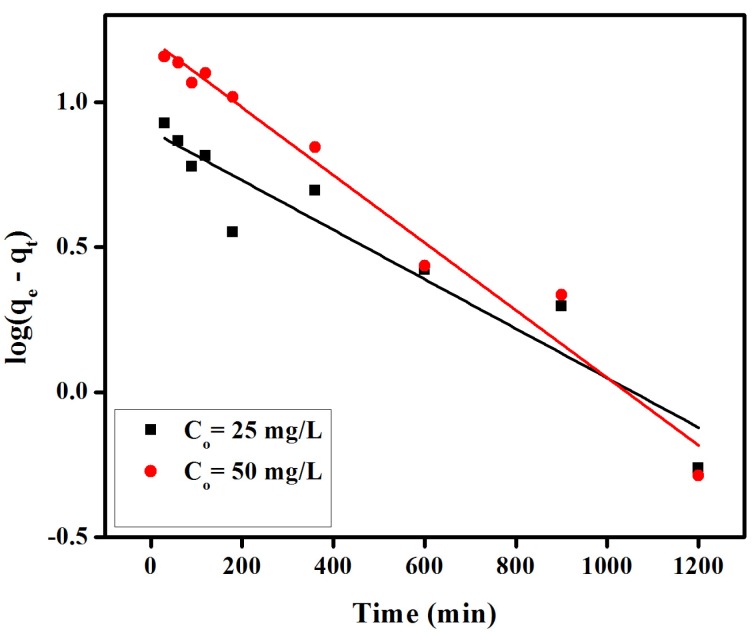
Pseudo-first-order Kinetics for adsorption of congo red on anion exchange membrane EBTAC.

In view of the proposed adsorption systems, several kinetic model studies were investigated to find out a suitable model to accurately describe the proposed adsorption system. These included pseudo-first-order and pseudo-second-order, liquid film diffusion and Elovich models. Starting from the pseudo-first-order model, the linearized form of the Lagergren pseudo-first-order rate equation is given by [34].

(1) $$loq(q_{e}-q_{t})=\log q_{e}-\frac{K_{1}t}{2.303}$$

where *q_e_* and *q_t_* is the amount of adsorbate adsorbed at equilibrium and time *t*, respectively, and *K*_1_ (min^−1^) is the rate constant of pseudo-first-order adsorption model. The plots of log(*q_e_* − *q_t_*) *vs.* time for pseudo-first-order model have been given in Figure 6. *K*_1_ values are calculated from the slope of Equation (1) and given in Table 1. These plots are linear, although the linearity of these curves does not necessarily ensure the mechanism due to the inherent disadvantage of correctly estimating equilibrium adsorption capacities [35]. A large difference was observed between experimental adsorption capacity value (*q_e_*, exp) and calculated adsorption capacity value (*q_e_*, cal), therefore pseudo-first-order model Kinetics as deemed as an inappropriate model to explain the proposed adsorption behavior.

**Figure 7:**
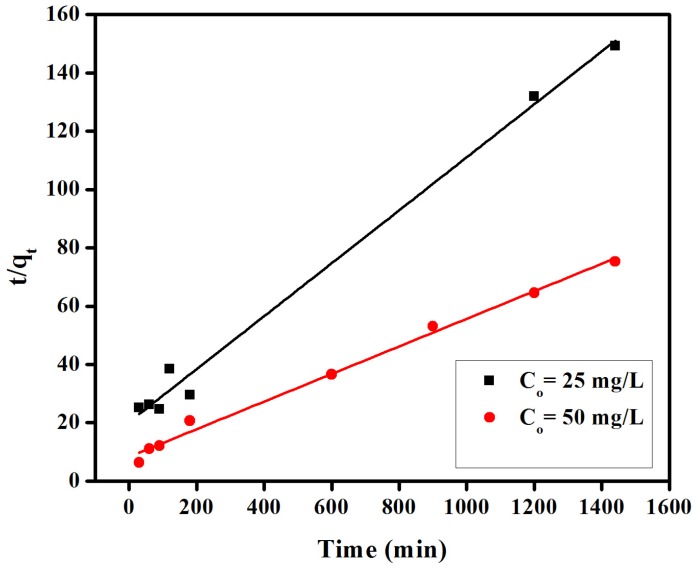
Pseudo-second-order Kinetics for adsorption of congo red on anion exchange membrane EBTAC.

Comparatively, the linearized form of pseudo-second kinetic model is expressed as [36].

(2) $$\frac{t}{q_{t}}=\frac{1}{k_{2}q_{e}^{2}}+\frac{t}{q_{e}}$$

where *k*_2_ (g mg^−1^ min^−1^) is the rate constant of pseudo-second-order model. The graphical representation of the pseudo-second-order model is depicted in Figure 7. The values of adsorption capacity (*q_e_*) corresponding to different initial dye concentrations could be determined from the slope of Figure 7 and given in Table 1. The adsorption capacity (*q_e_*) was observed to increase from 11.01 to 21.13 mg/g, with increasing initial dye concentrations from 25 to 50 mg/L. These results indicated dye removal from aqueous solution based on the initial dye concentration, with high values of correlation coefficient (R2 > 0.99), confirming a good fit of experimental data with a pseudo-second-order kinetic model.

**Table 1:** Kinetic parameters for the effect of concentration on the adsorption of CR onto anion exchange membrane (EBTAC).

Concentration (mg/L)	25	50
*q_e_*_(exp)_ (mg g^−1^)	9.64	19.12
Pseudo-first-order model		
*q_e_* (mg g^−1^)	7.46	16.41
k_1_ (×10^−3^ min^−1^)	0.85	1.20
R^2^	0.896	0.970
Pseudo-second-order model		
*q_e_* (mg g^−1^)	11.01	21.73
k_1_ (×10^−4^ g mg^−1^ min^−1^)	4.10	2.70
R^2^	0.991	0.992
Liquid film diffusion model		
K_fd_ (×10^−3^ min^−1^)	1.96	2.69
C_fd_	−0.193	−0.153
R^2^	0.897	0.970
Elovich model		
α (mg g^−1^ min^−1^)	8.94	3.74
β (g mg^−1^)	0.48	0.24
R^2^	0.917	0.951
The Bangham equation		
K_o_ (mL/(g/L))	0.53	0.91
α	0.48	0.40
R^2^	0.884	0.971
The modified Freundlich equation		
m	2.12	2.54
K (L/g min)	0.014	0.028
R^2^	0.883	0.971

Further investigations on liquid film diffusion, Elovich, Bangham and Freundlich kinetic models (see below) expressed as follow [37].

Liquid film:

(3) $$Ln(1-F)=-K_{fd}t$$

Elovich:

(4) $$q_{t}=\frac{1}{\beta }\ln(\alpha \beta )+\frac{1}{\beta }\ln t\;\text{[38]}$$

**Figure 8:**
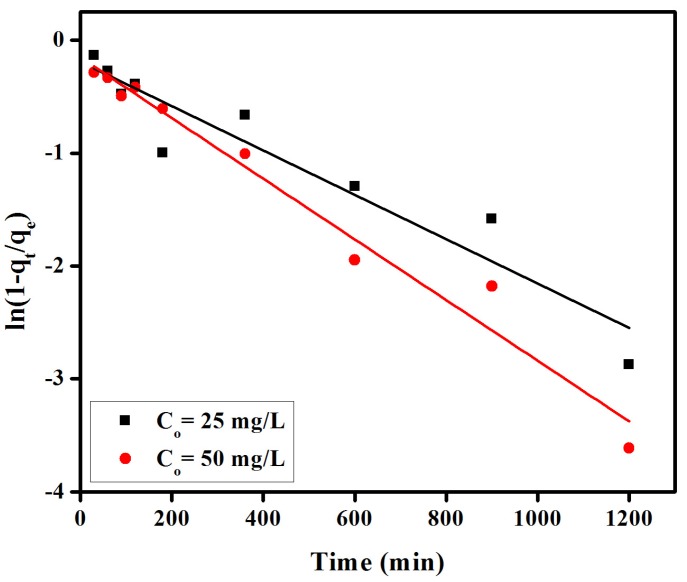
Liquid film diffusion model for adsorption of congo red on anion exchange membrane EBTAC.

**Figure 9:**
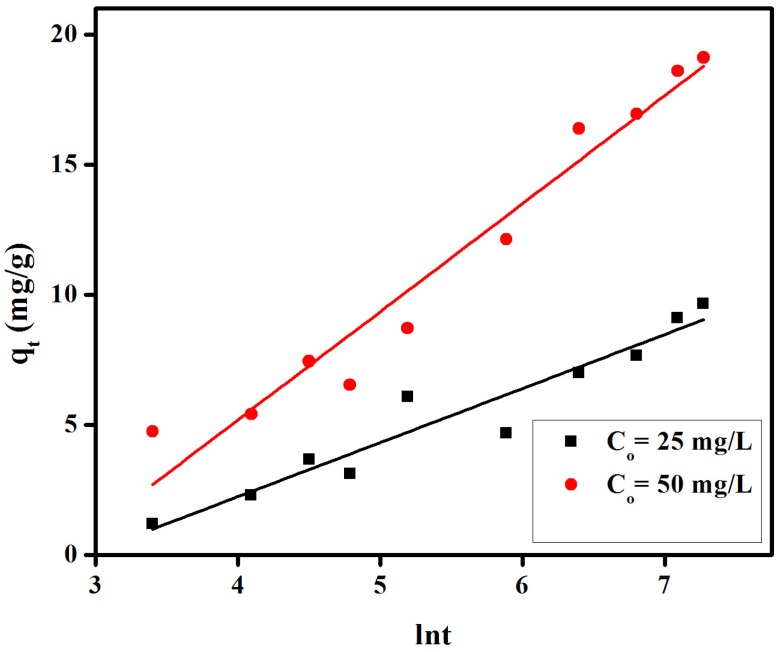
Elovich model for adsorption of congo red on anion exchange membrane EBTAC.

Bangham:

(5) $$\log\log\left(\frac{C_{o}}{C_{o}-q_{t}m}\right)=\log\left(\frac{k_{o}m}{2.303V}\right)+\alpha \log t$$

(6) $$\ln q_{t}=\ln\left(kC_{o}\right)+\frac{1}{m}\ln t\;\text{[39]}$$

provided values of correlation coefficients (R^2^) in the 0.898 to 0.970 range, inferior to those of a pseudo-second-order model, and thus unable to suitably explain the experimental data. All kinetic parameters for these models have been given in Figure 7, Figure 8, Figure 9 and Table 1.

**Figure 10:**
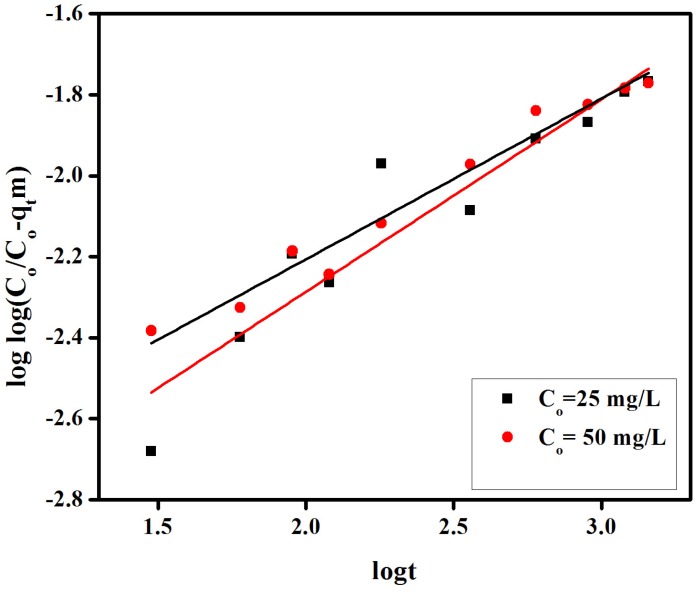
Banghamʼs plot of loglog(Co/Co-qtm) *vs.* logt for adsorption of congo red on anion exchange membrane EBTAC.

In the case of the Bangham equation, the plot of loglog(Co/Co-qtm) *vs.* logt for initial dye concentrations (25 mg/L and 50 mg/L, Figure 10) did not provide linear curves for the proposed CR removal indicating that the adsorbate diffusion into the pores of the membrane is not the only rate-controlling step [40]. Both film and pore diffusion may be important in different extension in the removal CR from aqueous solutions.

### 2.3. Adsorption Thermodynamics

To study the effect of temperature on the adsorption of CR on anion exchange membrane, experiments were conducted in the range of 293–323 K under optimized experimental conditions. Thermodynamic parameters indicate the feasibility and spontaneity of the adsorption process. Key parameters, namely enthalpy (*∆H°*) and entropy (*∆S°*) were determined by using the linear form of Van’t Hoff equation:

(7) $$\ln Kc=\frac{\Delta S^{o}}{R}-\frac{\Delta H^{o}}{RT}$$

The equilibrium constant “*K_c_*” was calculated by using the equation

(8) $$K_{c}=\frac{C_{a}}{C_{e}}$$

**Figure 11:**
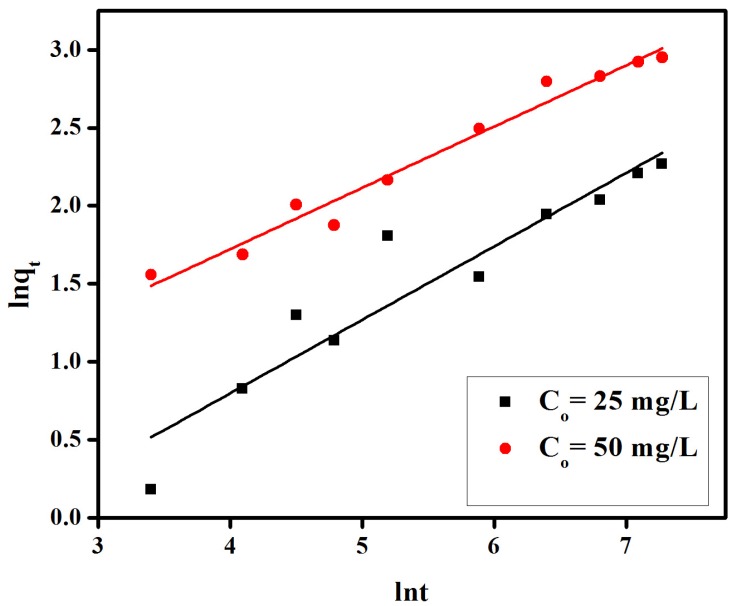
The modified Freundlich plot of ln_t_ *vs*. ln qt for adsorption of congo red on anion exchange membrane EBTAC.

**Figure 12:**
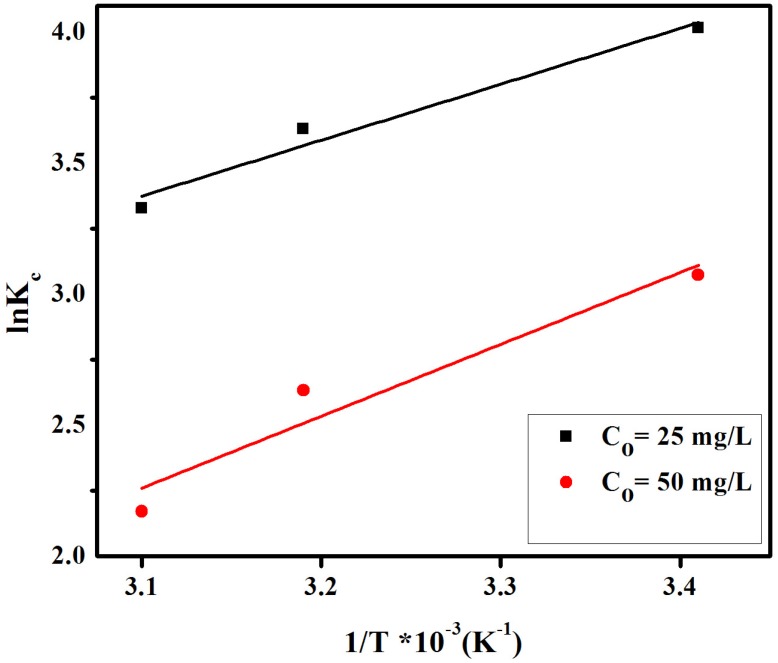
Plot of lnk_c_ verses 1/T for congo red dye on anion exchange membrane EBTAC.

The values of changes in Gibb’s free energy (*∆G°*) at different temperature were calculated by using following expression:

(9) $$\Delta G^{o}=\Delta H^{o}-T\Delta S^{o}$$

where *Kc*, *C_a_*, *C_e_*, *R*, *T* are the equilibrium constant, amount of dye (mol L^−1^) adsorbed on the adsorbent per liter (L) of the solution at equilibrium, equilibrium concentration (mol L^−1^) of dye in solution, general gas constant (0.008314 kJ mol^−1^ K^−1^) and absolute temperature (K), respectively. Similarly, *∆G°*, *∆H°* and *∆S°* stand for changes in Gibb’s free energy (KJ mol^−1^), enthalpy (KJ mol^−1^) and entropy (J mol^−1^ K^−1^), respectively. The plots of ln*Kc* verses 1/*T* for different initial dye concentration of 25 mg/L and 50 mg/L is shown in Figure 11 and Figure 12. The adsorption enthalpy (*∆H°*) and entropy (*∆S°*) were determined from slope and intercept of Figure 12 and summarized in Table 2. The values of Gibb’s free energy (*∆G°*) were positive for both initial dye concentrations of 25 mg/L and 50 mg/L at all studied temperatures. In both cases, such values increase at increasing temperatures as represented in Table 2. These findings may relate to an interaction between adsorbent and adsorbate, with unbalanced competition attributed to heterogeneities of the membrane surface. Negative values enthalpy (*∆H°*) indicate that the adsorption of CR under the investigated conditions is an exothermic process. Similarly, negative values of entropy (*∆S°*) represent a decrease in randomness at the dye-membrane interface during the adsorption process.

**Table 2:** Thermodynamic parameters for adsorption of CR on anion exchange membrane EBTAC.

C_o_ (mg/L)	∆H (KJ mol^−1^)	∆S (J mol^−1^)	∆G (KJ mol^−1^)		
			293 K	313 K	323 K
25	−17.70	−27.01	7.90	8.44	8.71
50	−22.76	−51.85	15.16	16.21	16.72

## 3. Experimental Section

### 3.1. Adsorbent

The commercial anion exchange membrane EBTAC was provided by Chemjoy Membrane Co. Ltd, Hefei, Anhui, China. The ion exchange capacity (IEC) and water uptake (W_R_) of EBTAC membrane are 0.42 mmol/g and 164.31%, respectively. EBTAC was used as adsorbant of anionic dye Congo (CR). The membrane was conditioned with 1 M HCl and NaOH to withdraw impurities prior to the experiments of this work.

### 3.2. Adsorbate

Congo red (Sodium salt of benzidinediazobis-1-nephthylamine-4-sulphonic acid) is a benzidine-based azo dye used as adsorbate in this study. The molecular formula of CR is C_32_H_22_N_6_Na_2_O_6_S_2_ and its molecular structure is shown in Figure 1. Congo red (CR) is mainly present in the effluents discharged from textile, paper, printing and leather industries [41] as well as during dyeing operation; about 15% of CR ends up in wastewaters [42]. All other reagents utilized in this work were of analytical grade and deionized water was used throughout the experiments. 

### 3.3. Adsorption

Batch adsorption of congo red (CR) dye was carried out by immersing the anion exchange membrane (EBTAC) into a measured volume of an aqueous solution containing CR at room temperature. Bottles were shaken at a constant speed of 120 rpm. The concentration of CR was determined by UV/vis spectrophotometer (UV-2550, SHIMADZU) and related calibration curves were obtained. The wavelength used for CR was 490 nm. CR adsorption on membrane at time t, was calculated by Equation (10).

(10) $$q_{t}=\frac{C_{o}-C_{t}}{W}\times V$$

where *C_o_* and *C_t_* are the concentration of CR at initial stage and at time *t*, respectively. Similarly, *V* and *W* are volume of CR aqueous solution and weight of adsorbent respectively.

## 4. Conclusions

This contribution was aimed at providing some fundamental studies on the adsorption of anionic dyes (CR) from aqueous solutions using EBTAC at different initial dye concentrations. The influences of contact time, membrane dosage, ionic strength, and temperature on the removal of CR were studied. These results revealed that CR removal increases at increasing contact time and membrane dosage (being two critical parameters) and comparably decreases with an increase in ionic strength and temperature (with the latter not having a significant influence in adsorption properties). Adsorption Kinetics showed that the experimental data fitted well with a pseudo-second-order model and thermodynamic studies further confirmed that the adsorption of CR on anion exchange membrane under the investigated conditions was an exothermic process. In conclusion, the use of anion exchange membranes can offer a promising alternative adsorbent for CR dye removal from wastewater, with further studies ongoing to apply similar principles to other dyes as well as actual wastewater effluents. Future work will be carried out with actual wastewater from dyeing processes to compare with the simulated dye solutions, particularly as additives may affect the efficacy of absorption. Additionally, scale up factors need to be investigated prior to practical benefits of the use in dye works can be substantiated.

## Nomenclatures

CR: congo red

*q_t_*: Adsorption capacity at time “t”

*q_e_*: Adsorption capacity at equilibrium

IEC: Ion exchange capacity

WR: Water uptake

*∆G°*: Change in Gibb’s energy

*∆S°*: Change in entropy

*∆H°*: Change in enthalpy
